# Mobile App for Gynecologic Cancer Support for Patients With Gynecologic Cancer Receiving Chemotherapy in China: Multicenter Randomized Controlled Trial

**DOI:** 10.2196/49939

**Published:** 2023-11-13

**Authors:** Huicong Lin, Mingzhu Ye, Yanjuan Lin, Fuhong Chen, Sally Chan, Hongxia Cai, Jiemin Zhu

**Affiliations:** 1 School of Medicine Xiamen University Xiamen, Fujian Province China; 2 Women and Children's Hospital School of Medicine Xiamen University Xiamen, Fujian Province China; 3 Department of Gynecology and Obstetrics Zhongshan Hospital Xiamen University Xiamen, Fujian Province China; 4 Department of Nursing Fujian Medical University Union Hospital Fuzhou, Fujian Province China; 5 Department of Nursing First Affiliated Hospital Xiamen University Xiamen, Fujian Province China; 6 President Office Tung Wah College Hongkong China; 7 Department of Gynecology and Obstetrics First Affiliated Hospital Xiamen University Xiamen, Fujian Province China; 8 Department of Nursing Women and Children's Hospital School of Medicine, Xiamen University Xiamen China; 9 Cancer Care Research Unit Faculty of Medicine and Health, Susan Wakil School of Nursing and Midwifery The University of Sydney Sydney Australia

**Keywords:** mobile app, gynecologic cancer, uncertainty in illness, quality of life, symptom distress, social support, cancer care, gynecologic, application, chemotherapy, support, women, well-being, emotional well-being, cancer, mobile phone

## Abstract

**Background:**

Patients with gynecologic cancer receiving chemotherapy often report unmet supportive care needs. Compared with traditional face-to-face clinical interventions, mobile health can increase access to supportive care and may address patients’ needs. Although app-based support programs have been developed to support patients with gynecologic cancer, their efficacy has not been adequately tested.

**Objective:**

The aim of this study was to examine the efficacy of a mobile app for gynecologic cancer support (MGCS) for patients with gynecologic cancer receiving chemotherapy in China.

**Methods:**

A multicenter randomized controlled trial was conducted in 2 university-affiliated hospitals in China. A total of 168 Chinese patients with gynecologic cancer were recruited and randomized to receive routine care or MGCS program plus routine care for 24 weeks. The Mishel uncertainty in illness theory guided the development of MGCS program, which has 4 modules: weekly topics, emotional care, discussion center, and health consultation. The primary outcome of this program was the assessment of the uncertainty in illness. The secondary outcomes were quality of life, symptom distress, and social support. All health outcomes were evaluated at baseline (T0), 12 weeks (T1), and 24 weeks (T2). Repeated measures analysis of covariance was used to assess the efficacy of the MGCS program.

**Results:**

In this trial, 67 patients in the control group and 69 patients in the intervention group completed 2 follow-up assessments (response rate, 136/168, 81%). At 12 weeks, no significant differences were observed in any of the health outcomes between the 2 groups. At 24 weeks, compared to patients in the control group, those in the intervention group reported significant decreased uncertainty in illness (*P*<.001; *d*=–0.60; adjusted mean difference –7.69, 95% CI –11.31 to –4.07) and improved quality of life (*P*=.04; *d*=0.30; adjusted mean difference 4.77, 95% CI 0.12-9.41).

**Conclusions:**

The MGCS program demonstrated efficacy in supporting patients with gynecologic cancer receiving chemotherapy. This trial illustrates that an app-based program can be incorporated into routine care to support patients with cancer and suggests that allocation of more resources (grants, manpower, etc) to mobile health in clinics is warranted.

**Trial Registration:**

Chinese Clinical Trial Registry ChiCTR2000033678; https://www.chictr.org.cn/showproj.html?proj=54807

## Introduction

### Background

Gynecologic cancer (GC) is a global public health concern, with cervical, endometrial, and ovarian cancers being the 3 most common GCs [1]. In China, their incidence rates ranked in the top 10 cancers for women in 2020 [2]. Chemotherapy is commonly used to treat GC [3]. The diagnosis of GC and the corresponding chemotherapy have adverse effects on patients’ fertility, sexuality, identity, and body image [4]. Patients with GC receiving chemotherapy often report unmet supportive care needs [5], which may increase their risks of physical and psychological morbidity, leading to high risk of recurrence, shorter survival, and poor prognosis [6,7]. Uncertainty of illness, information needs, symptom management strategies, and counselling services were identified as the common supportive care needs for this growing population [5,8]. Compared with traditional face-to-face interventions, web-based support programs may overcome the long-distance and time barriers to better address patients’ supportive care needs [9]. In 2021, over 1.028 billion Chinese individuals, accounting for 73% of the Chinese population, accessed the internet through their mobile phones [10]. Mobile apps may provide a promising and easily accessible support platform to reach the large cancer population. Although app-based support programs have been previously developed to support patients with GC, their efficacy has not been adequately demonstrated [11].

### Conceptual Framework

Many patients with GC receiving chemotherapy experience high levels of uncertainty in illness [12]. According to Mishel uncertainty in illness theory, uncertainty in illness occurs when an individual is not able to assign a definite meaning to an illness-related event or to accurately determine potential illness outcomes [13]. Uncertainty can stem from ambiguity and complexity. Ambiguity refers to an individual’s lack of credible or sufficient information regarding the disease prognosis and corresponding treatments [14]. Complexity means that a variety of risk factors (eg, treatment outcomes, side effects) make an illness more difficult to understand [14]. Additionally, uncertainty in illness is positively related to unfamiliar treatment-related symptom distress [15] and inadequate social support [16]. Increased uncertainty in illness has been shown to affect patients’ quality of life (QoL) [17]. A literature review investigated the application of Mishel uncertainty in illness theory for people with cancer and found that a variety of interventions have been developed, which featured uncertainty components such as antecedent and appraisal of uncertainty, coping, communication strategies, information provision, and reframing [18]. These interventions primarily targeted uncertainty, which ultimately promote QoL for people with cancer [18]. However, there was no intervention that applied Mishel theoretical frameworks for patients with GC.

We developed the mobile app for gynecologic cancer support (MGCS) program under the conceptualization of Mishel uncertainty in illness theory. The primary aim of this trial was to examine whether the MGCS program can effectively target uncertainty in illness. We hypothesized that MGCS plus routine care is superior to routine care alone in reducing uncertainty in illness. The subhypotheses were that MGCS plus routine care would improve QoL, reduce symptom distress, and promote social support for patients with GC.

## Methods

### Study Design

A multicenter randomized controlled trial was conducted. This trial was registered in the Chinese Clinical Trial Registry (ChiCTR2000033678). The protocol for this trial was published and strictly followed at all stages [19]. Findings are presented following the guidelines of CONSORT-EHEALTH (Consolidated Standards of Reporting Trials of Electronic and Mobile Health Applications and Online Telehealth) (Multimedia Appendix 1) [20].

### Participants and Settings

Trial participants included patients who were (1) aged 18 years and older, (2) first diagnosed with GC (including cervical, endometrial, and ovarian cancers), (3) starting the first cycle of chemotherapy, (4) able to access the internet via a mobile phone, (5) contactable, and (6) able to read and write Chinese. Patients were excluded if they had (1) other major health conditions, including other cancers and (2) other major mental health conditions, which may prevent them from participating in the MGCS program. Patients were excluded if they were not aware of their diagnoses of a GC. Although not common, this does occur in China when families seek to protect patients from bad news. Participants were recruited from the Department of Gynecology in 2 Chinese university-affiliated hospitals. The hospitals administer chemotherapy to approximately 500 (400 and 100 in each hospital, respectively) patients with GC each year. Patients with GC need to stay overnight in the hospital on the first day of each cycle of chemotherapy. Approximately 50% of patients with GC were eligible to participate in this trial.

### Sample Size Calculation and Sample Randomization

The primary health outcome of uncertainty was used to calculate the sample size. Previous research on a face-to-face uncertainty management intervention reduced uncertainty in illness for patients with HIV with an effect size of 0.53 [21]. Another study reported that a mobile navigation program reduced uncertainty in illness for patients with colorectal cancer with an effect size of 0.49 [22]. Thus, the effect size (0.49) of web-based intervention for patients with cancer was used to determine the sample size. A total of 134 participants was needed, with a power of .80 and a significance level of .05. A 20% dropout rate was expected; therefore, the eventually adjusted sample size for our study was 168 in total. The web-based research randomizer was used, and the blocked design was chosen to meet the desirable equal allocations in 2 groups [23]. A block size of 4 was chosen, and the block size was kept blind from the investigator to avoid selection bias [24]. For each participating hospital, participants were randomized to the intervention group or the control group to ensure an allocation ratio of 1:1.

### Ethics Approval

Ethics approval was obtained from the ethics committees of the School of Medicine in Xiamen University (XDYX2019013) and 2 university-affiliated hospitals (Zhongshan Hospital: XMZSYYKY2020-113 and First Affiliated Hospital: FA2020-037). All participants were informed that their participation was voluntary. Participants needed to complete informed consent before participation. All collected data were kept confidential and anonymous. Participants received a gift compensation (approximately US $4) after each evaluation.

### Intervention

A health care team consisting of 3 gynecologic oncologists, 1 psychologist, and 2 oncology nursing specialists designed the MGCS program. An information technology company technically developed and subsequently maintained the MGCS program. The Mishel theory outlines 4 main components of uncertainty in illness: antecedents (stimuli frame and structure providers), appraisal (danger and opportunity), coping, and adaptation [18]. Antecedents (eg, unfamiliar illness-related stimuli, low social support) may generate uncertainty, and thereafter, an individual appraises the uncertainty as a danger or an opportunity. Appraising the uncertainty as a danger, the individual adopts affect-control (such as emotional support) and mobilizes (such as seeking information) coping strategies to minimize uncertainty. Appraising the uncertainty as an opportunity, the individual adopts buffering coping strategies (such as reframing) to manage uncertainty. If these coping strategies are effective, the individual can adapt to the illness.

The MGCS program has 4 modules: weekly topics, emotional care, discussion center, and health consultation [19]. The information resources of the weekly topics were derived from the website of American Cancer Society [25]. The weekly topics module targets the antecedents, appraisal, and coping of uncertainty by providing credible information to decrease unfamiliar cancer-related stimuli and help the patient appropriately appraise and cope with their illness and treatment side effects, thus decreasing ambiguity. The emotional care module targets coping by providing relaxation and distraction strategies, which may help manage the emotional response to diagnosis and treatment to decrease the complexity. The discussion center module offers emotional and informational support from peers, and the health consultation module offers tailored advice from the health care team to help the patient manage uncertainty. Communicating with peers or health care team targets the antecedent variables of structure providers (social support and credible authority), appraisal, and coping of uncertainty with the purpose to decrease both ambiguity and complexity. The details of the 4 modules of the MGCS program are described in Table 1.

**Table 1:** Four modules of the mobile app for gynecologic cancer support program.

Module	Multimedia channel of delivery	Notification	Main content	Targeted aspects of Mishel uncertainty in illness theory
Weekly topics	Text, graphics, images, videos, and emoticon pictures	Notifications on newly available information were sent to participants.	A total of 24 topics were developed, with 1 new topic automatically uploaded each week. The 24 topics coincide with the illness’ treatment course: (1) information on cancer, (2) cancer treatments, (3) summary of treatment-induced side effects, (4) survival rates, (5) infection prevention, (6) uncertainty management, (7) emotion regulation, (8) alopecia, (9) nausea and vomiting, (10) dry mouth, (11) appetite, (12) constipation, (13) fatigue, (14) peripheral neuropathy, (15) sleep problems, (16) sweats, (17) cancer pain, (18) sexual problems, (19) exercise, (20) communication skills, (21) chemotherapy resistance, (22) information on recurrence, (23) management of recurrence, and (24) cancer survivorship. Participants could provide comments or reflections at the bottom of each topic after reading the topics.	The antecedents, appraisal, and coping uncertainty
Emotional care	Text, image, and audio	Notifications on newly available information were sent to participants.	Relaxation and distraction strategies (soothing music, lighthearted stories, and exercise plans) were provided and updated every week until week 24.	Coping uncertainty
Discussion center	Text and emoticon pictures	Icon of discussion center flickered when a new message was posted.	The discussion center was moderated by the researchers. Participants could share their experiences and text with others. Further, the researchers (HL, MY, and YL) read all messages each day, encouraged participants to share their experiences, and provided appropriate advice when requested.	The antecedents and coping uncertainty
Health consultation	Text and emoticon pictures	When a new question was posted, the icon of health consultation flickered for the health care team. When a response was posted, the icon of health consultation flickered for the woman who asked the question.	When a woman asked a health-related question, any member of the health care team could respond. Responses were sent within 24 hours. To protect privacy, the question and the response were only accessible by the individual who posted the question and the health care team.	The antecedents, appraisal, and coping uncertainty

Different strategies were provided to make MGCS content easy to understand, such as the use of graphics, images, short videos, emoticon pictures, and plain language. The perceived usefulness and perceived ease of use of the MGCS program were assessed by 8 patients with GC receiving chemotherapy, and they all found that this program provided useful information and was easy to use. No major components and contents were changed during the implementation of the program.

Participants in the intervention group were given a QR code to download the MGCS onto their mobile phones (Android and iOS versions available). Once access to the app was approved by the researchers, participants could use their mobile phone number and a self-set password to access the MGCS program when and where needed. The researchers offered 30-minute individual face-to-face training to participants on how to use the 4 modules. Training included assisting participants to explore the content in the modules of weekly topic and emotional care as well as to write their first message in the modules of discussion center and health consultation. When participants logged into the app for the first time, a tutorial guide video was displayed initially. Once viewed, the video was automatically saved in the tutorial section of the individual center for future reference. Participants could seek technical assistance in the help section in the individual center. Access to the app automatically expired 24 weeks after activation.

Participants in both groups received routine care from health care workers during their stay in the hospitals for each cycle of chemotherapy. Before the first chemotherapy cycle, the patient’s oncologist offered information regarding chemotherapy and chemotherapy-related side effects. The ward nurse provided written information on how to manage the side effects of chemotherapy and answered any question. Currently, there are no app-based programs to help patients with GC to cope with uncertainty and the side effects of chemotherapy at the participating hospitals.

### Outcome Measures

Self-reported demographic and clinical characteristics questionnaires were designed by the research team. Demographic characteristics consist of age, marital status, educational level, current employment, monthly family income (in USD), and payment methods. Clinical characteristics include primary disease site, stage of cancer, treatment before chemotherapy, cycles of chemotherapy, and chemotherapy regimen. Clinical characteristics were confirmed through the participants’ electronic medical records.

### Primary Outcome

Participants’ uncertainty in illness was assessed with the Chinese version of the 25-item Mishel Uncertainty in Illness Scale for Adults (MUIS-A) [26]. The MUIS-A has been widely used to assess uncertainty in illness in patients with cancer [27]. The MUIS-A Chinese version has demonstrated excellent content validity (content validity index=0.97) and good internal consistency (Cronbach α=.90) [28]. The MUIS-A has 2 subscales: ambiguity and complexity. A higher total score indicates greater uncertainty in illness (scores ranging from 25 to 125). The MUIS-A (Cronbach α=.91) exhibited good baseline internal consistency in this trial.

### Secondary Outcomes

Participants’ QoL was measured with the Chinese version of the 27-item Functional Assessment of Cancer Therapy-General (FACT-G, version 4) [29]. The FACT-G has been used to evaluate the QoL of patients with GC across different cultures [30,31]. The FACT-G Chinese version has been proven as a valid instrument, with Cronbach α of .92 [32]. The FACT-G has 4 subscales: physical, social or family, emotional, and functional well-being. A higher total score shows better QoL (scores ranging from 0 to 108). The FACT-G (Cronbach α=.89) exhibited good baseline internal consistency in this trial. Participants’ symptom distress was evaluated with the Chinese version of the 19-item MD Anderson Symptom Inventory (MDASI) [33]. The MDASI has been widely used to evaluate symptom distress among patients with GC [34]. The Chinese version of MDASI has been applied to assess symptom distress among patients with breast cancer and displayed good internal reliability [35]. The mean score ranges from 1 to 10, with a higher score indicating greater symptom distress. The MDASI (Cronbach α=.91) exhibited good baseline internal consistency in this trial. Participants’ social support was assessed with the Chinese version of the 12-item Multidimensional Scale of Perceived Social Support (MSPSS) [36]. The MSPSS has been widely applied to evaluate the perceived social support of patients with cancer [37]. The validity of the MSPSS Chinese version has been extensively demonstrated (Cronbach α=.89) [35]. The mean score ranges from 1 to 7, with higher scores indicating better social support. The MSPSS (Cronbach α=.92) exhibited good baseline internal consistency in this trial. The 24 weeks’ usage data (log-in frequency and duration) of each module and the whole MGCS program was recorded in a background thread.

### Data Collection

Data were collected between February 2021 and March 2022. The oncologist informed eligible patients with GC about the MGCS program. Patients with GC who expressed an interest in this study were approached by a member of the research team to provide more detailed information. After signing the paper consent form, all participants completed paper-based demographic and baseline health outcomes questionnaires before randomization (T0). Participants were notified of their group allocations in the trial. Following the protocol of the participating hospitals, patients with GC received 4-8 cycles of chemotherapy (3 weeks/cycle). Chemotherapy was usually completed within 24 weeks. Participants in both groups completed the paper questionnaires at 12 (T1) and 24 weeks (T2). The researchers who collected questionnaires were not blind to group allocation. If the participants did not return to the hospital at either time point, a researcher contacted them by phone and mailed them the questionnaires.

### Statistical Analysis

SPSS (version 25; IBM Corp) was applied to analyze all the data [38]. To avoid attrition bias, intention-to-treat analysis was performed to manage the missing data at T1 and T2 with the last observation carried forward method. Independent sample 2-sided *t* tests and chi-square tests (or Fisher exact test) were used to examine the continuous or categorical variables between the 2 groups at baseline. The statistical assumption of analysis of covariance is that the covariates are correlated with the dependent variable [39]. The demographic or clinical variables (including age and cancer stage) were not added as covariates because no association was found between these variables and the primary outcome (uncertainty in illness at T1). The MGCS program’s effects on uncertainty, QoL, symptom distress, and social support were examined using repeated measures analysis of covariance with time as a within-subject factor, a group as a between-subject factor, and the time × group interaction, adjusted for baseline corresponding health outcome. In the covariance analysis, multivariate tests were performed when Mauchly test of sphericity was not satisfied. The adjusted mean difference (95% CI) was reported, with the unadjusted and adjusted mean, significance level, and effect size (Cohen *d*).

## Results

### Participants’ Characteristics

**Figure 1:**
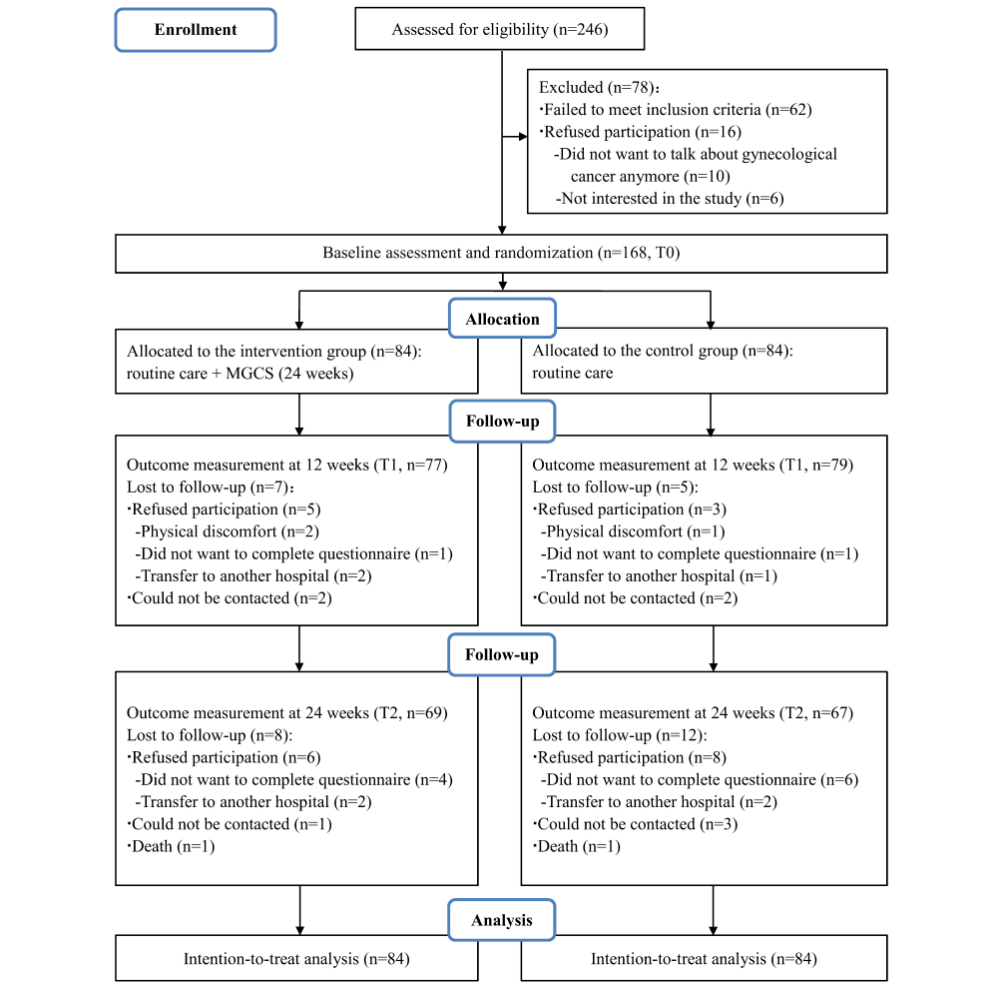
CONSORT (Consolidated Standard of Reporting Trials) diagram of the mobile app for gynecologic cancer support program. MGCS: mobile app for gynecologic cancer support; T1: after 12 weeks; T2: after 24 weeks.

Participants were recruited between February and October 2021. Of the 246 participants approached by the researchers, 43 were not able to read or write in Chinese, 11 were unaware of the GC diagnosis, 6 had concurrent cancers, and 2 had concurrent mental health problems; thus, 62 participants did not meet the inclusion criteria and 16 participants declined to participate after receiving detailed information on the program. Finally, 168 participants completed baseline assessment and randomization. At T1, 12 participants (7 in the intervention group and 5 in the control group) withdrew from the study. At T2, a further 20 (8 in the intervention group and 12 in the control group) withdrew from this study. No significant difference in the dropout rate existed between the 2 groups (*P*=.36). Finally, 69 participants in the intervention and 67 participants in the control group completed all follow-up assessments (response rate, 136/168, 81%). Figure 1 shows the trial’s flowchart using the CONSORT guidelines [40]. More than half of the participants (86/168, 51.2%) were aged between 45 and 59 years. Most participants had a diagnosis of either ovarian cancer (67/168, 39.9%), cervix uteri cancer (65/168, 38.7%), or corpus uteri cancer (34/168, 20.2%). Most of the participants were in stage I-III of cancer (stage I, 52/168, 31%; stage II, 38/168, 22.6%; stage III: 61/168, 36.3%). Of the 168 participants, 112 (66.7%) underwent surgery prior to commencing adjuvant chemotherapy. Most participants (142/168, 84.5%) received 4-8 cycles of chemotherapy. No significant differences (*P*>.05) were found between the 2 groups at baseline in terms of demographic and clinical characteristics and measurements (Table 2).

**Table 2:** Demographic and clinical characteristics and measurements at baseline of the intervention and control groups (N=168).

Variables		Total (N=168)	Intervention group (n=84)	Control group (n=84)	*t*/*χ*^2^*(df)* or Fisher exact test		*P* value	
**Age (years), n (%)**						6.05 (2)^a^		.05
	<45	52 (31)	33 (39.3)	19 (22.6)				
	45-59	86 (51.2)	36 (42.9)	50 (59.5)				
	≥60	30 (17.9)	15 (17.9)	15 (17.9)				
**Marital status, n (%)**						0.08 (1)^a^		>.99
	Single/divorced/separated/widowed	13 (7.7)	6 (7.1)	7 (8.3)				
	Married/cohabitation	155 (92.3)	78 (92.9)	77 (91.7)				
**Educational level, n (%)**						0.23 (1)^a^		.75
	Junior high school or below	105 (62.5)	51 (60.7)	54 (64.3)				
	Senior high school or above	63 (37.5)	33 (39.3)	30 (35.7)				
**Current employment, n (%)**						0.14 (1)^a^		.85
	Employed	38 (22.6)	20 (23.8)	18 (21.4)				
	Unemployed/retired	130 (77.4)	64 (76.2)	66 (78.6)				
**Monthly family income (USD), n (%)**						2.68 (1)^a^		.14
	<1267	112 (66.7)	51 (60.7)	61 (72.6)				
	≥1267	56 (33.3)	33 (39.3)	23 (27.4)				
**Payment methods of health care costs, n (%)**						N/A^b^		.44
	Fully or partially covered by medical insurance	161 (95.8)	82 (97.6)	79 (94)				
	Self-paying	7 (4.2)	2 (2.4)	5 (6)				
**Primary disease site, n (%)**						2.85^c^		.41
	Cervix uteri	65 (38.7)	32 (38.1)	33 (39.3)				
	Ovary	67 (39.9)	37 (44)	30 (35.7)				
	Corpus uteri	34 (20.2)	15 (17.9)	19 (33.6)				
	Mixed types^d^	2 (1.2)	0 (0)	2 (2.4)				
**Stage of cancer, n (%)**						8.24^c^		.07
	Stage I	52 (31)	33 (39.3)	19 (22.6)				
	Stage II	38 (22.6)	19 (22.6)	19 (22.6)				
	Stage III	61 (36.3)	24 (28.6)	37 (44)				
	Stage IV	15 (8.9)	8 (9.5)	7 (8.3)				
	Mixed types^d^	2 (1.2)	0 (0)	2 (2.4)				
**Treatment before chemotherapy, n (%)**						4.37^c^		.37
	No	43 (25.6)	20 (23.8)	23 (27.4)				
	Surgery	112 (66.7)	59 (70.2)	53 (63.1)				
	Radiotherapy	3 (1.8)	1 (1.2)	2 (2.4)				
	Surgery + radiotherapy	6 (3.6)	1 (1.2)	5 (6)				
	Surgery + Traditional Chinese medicine treatment	4 (2.4)	3 (3.6)	1 (1.2)				
**Cycles of chemotherapy, n (%)**						1.33^c^		.67
	3 cycles or less	25 (14.9)	11 (13.1)	14 (16.7)				
	4-8 cycles	142 (84.5)	72 (85.7)	70 (83.3)				
	9 cycles or more	1 (0.6)	1 (1.2)	0 (0)				
**Chemotherapy regimen, n (%)**						4.84^c^		.58
	Paclitaxel + Cisplatin	20 (11.9)	7 (8.3)	13 (15.5)				
	Paclitaxel + Carboplatin	109 (64.9)	58 (69)	51 (60.7)				
	Bleomycin + Etoposide + Cisplatin	8 (4.8)	4 (4.8)	4 (4.8)				
	Docetaxel + Carboplatin	17 (10.1)	7 (8.3)	10 (11.9)				
	Doxorubicin + Carboplatin	5 (3)	2 (2.4)	3 (3.6)				
	Ifosfamide + Epirubicin	4 (2.4)	2 (2.4)	2 (2.4)				
	Others	5 (3)	4 (4.8)	1 (1.2)				
Mishel Uncertainty in Illness Scale for Adults, mean (SD)		70.27 (12.34)	70.88 (11.55)	69.67 (13.13)	–0.64 (166)^e^		.53	
Functional Assessment of Cancer Therapy-General, mean (SD)		67.30 (15.85)	67.03 (14.36)	67.58 (17.28)	0.23 (166)^e^		.82	
MD Anderson Symptom Inventory, mean (SD)		2.92 (1.68)	2.98 (1.88)	2.86 (1.46)	–0.44 (166)^e^		.66	
Multidimensional Scale of Perceived Social Support, mean (SD)		5.44 (0.85)	5.37 (0.78)	5.51 (0.92)	1.08 (166)^e^		.28	

^a^Chi-square test.

^b^N/A: not applicable. No value is provided for Fisher exact test in a 2×2 contingency table.

^c^Fisher exact test. No degrees of freedom are used in Fisher exact test.

^d^Mixed types (n=2): one participant was diagnosed with primary stage III ovarian cancer and primary stage I corpus uteri cancer, and the other one was diagnosed with primary stage I cervix uteri cancer and primary stage I ovarian cancer.

^e^Independent 2-sided *t* test value.

### Efficacy of the MGCS Program

#### Primary Outcomes

At 12 weeks (T1), no significant difference was found in uncertainty in illness between the 2 groups. At 24 weeks (T2), after adjusting for the baseline uncertainty in illness, participants who received the MGCS program plus routine care—compared with participants who received routine care only—exhibited a significant decrease in uncertainty in illness (*P*<.001; *d*=–0.60; adjusted mean difference –7.69, 95% CI –11.31 to –4.07; Table 3) [41].

**Table 3:** Effects of the mobile app for gynecologic cancer support program on primary and secondary health outcomes at 12 weeks (T1) and 24 weeks (T2) with intention-to-treat analysis (N=168)^a^.

Intervention effect			Intervention group (n=84), mean (SD/SE)	Control group (n=84), mean (SD/SE)	Mean difference (95% CI)	*P* value	Effect size (Cohen *d*)^b^
**Primary outcomes**							
	**Mishel Uncertainty in Illness Scale for Adults**						
		T1 (unadjusted)	63.04 (11.43)	64.30 (13.93)	–1.26 (–5.02 to 2.50)	.51	–0.10
		T1 (adjusted)	62.74 (1.23)	64.60 (1.23)	–1.86 (–5.29 to 1.57)	.29	–0.15
		T2 (unadjusted)	56.87 (13.20)	64.07 (12.47)	–7.20 (–11.20 to –3.20)	.001	–0.56
		T2 (adjusted)	56.63 (1.30)	64.32 (1.30)	–7.69 (–11.31 to –4.07)	<.001	–0.60
**Secondary outcomes**							
	**Functional Assessment of Cancer Therapy-General**						
		T1 (unadjusted)	70.52 (16.05)	69.78 (17.02)	0.74 (–4.47 to 5.96)	.78	0.04
		T1 (adjusted)	70.67 (1.54)	69.63 (1.54)	1.05 (–3.25 to 5.34)	.63	0.06
		T2 (unadjusted)	75.47 (15.50)	70.89 (16.73)	4.58 (–0.85 to 10.01)	.10	0.28
		T2 (adjusted)	75.57 (1.66)	70.80 (1.66)	4.77 (0.12 to 9.41)	.04	0.30
	**MD Anderson Symptom Inventory**						
		T1 (unadjusted)	2.96 (1.74)	3.09 (1.76)	–0.13 (–0.67 to 0.42)	.65	–0.07
		T1 (adjusted)	2.93 (0.16)	3.12 (0.16)	–0.19 (–0.64 to 0.26)	.41	–0.11
		T2 (unadjusted)	2.23 (1.62)	2.46 (1.88)	–0.23 (–0.81 to 0.35)	.43	–0.13
		T2 (adjusted)	2.21 (0.18)	2.49 (0.18)	–0.28 (–0.77 to 0.21)	.26	–0.16
	**Multidimensional Scale of Perceived Social Support**						
		T1 (unadjusted)	5.18 (0.95)	5.44 (0.82)	–0.26 (–0.53 to 0.02)	.06	–0.29
		T1 (adjusted)	5.21 (0.09)	5.41 (0.09)	–0.19 (–0.44 to 0.05)	.12	–0.23
		T2 (unadjusted)	5.22 (0.88)	5.41 (0.83)	–0.20 (–0.48 to 0.09)	.18	–0.22
		T2 (adjusted)	5.25 (0.09)	5.39 (0.09)	–0.14 (–0.39 to 0.10)	.25	–0.16

^a^Repeated measures multivariate analysis of covariance was used to examine the efficacy of the mobile app for gynecologic cancer support program on primary and secondary health outcomes at 12 weeks (T1) and 24 weeks (T2), adjusted by baseline corresponding health outcomes.

^b^Adjusted effect sizes were calculated by dividing the between-group difference of the postintervention means (adjusted for baseline values) by the pooled standard deviation [41].

#### Secondary Outcomes

**Figure 2:**
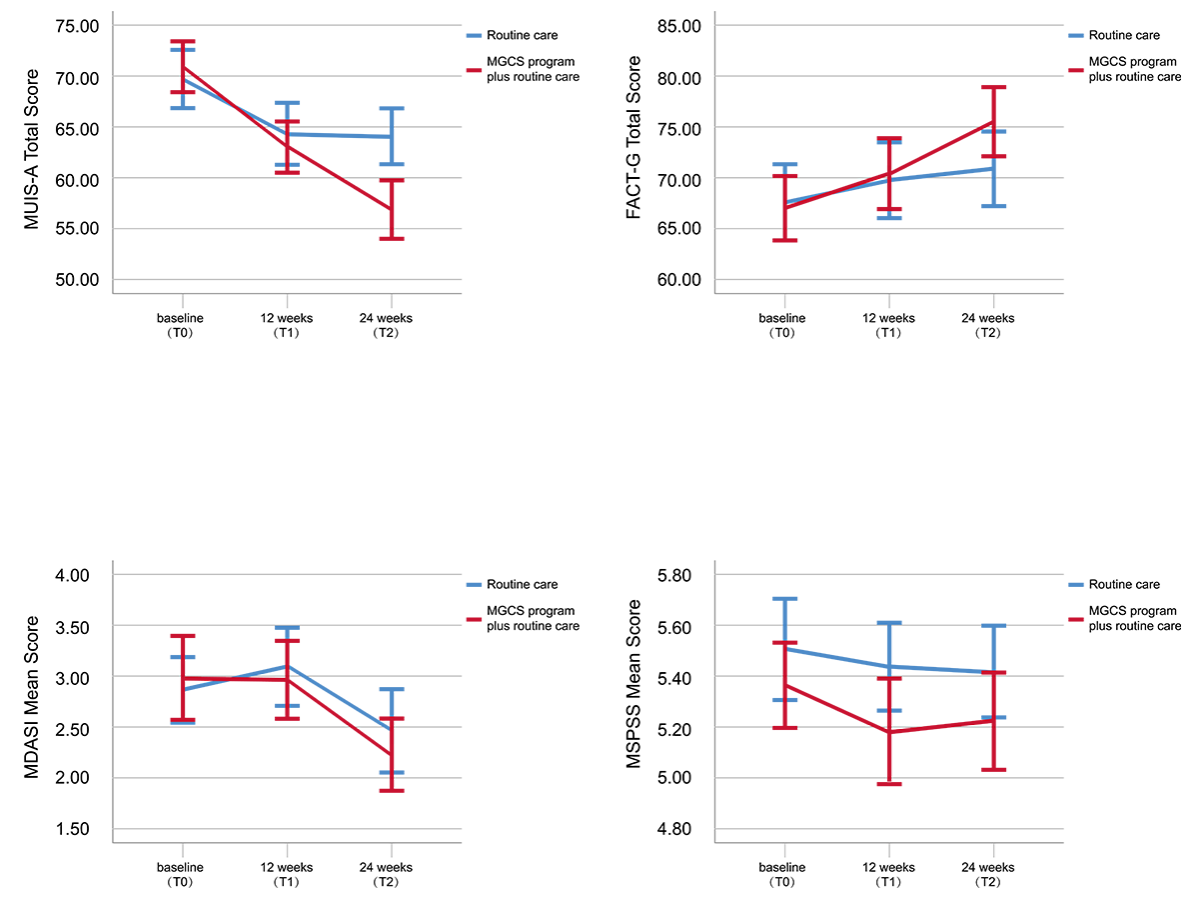
Mean change in the measurement outcomes at baseline (T0), 12 weeks (T1), and 24 weeks (T2) (N=168). FACT-G: Functional Assessment of Cancer Therapy-General; MDASI: MD Anderson Symptom Inventory; MGCS: mobile app for gynecologic cancer support; MSPSS: Multidimensional Scale of Perceived Social Support; MUIS-A: Mishel Uncertainty in Illness Scale for Adults.

At 12 weeks (T1), there was no significant difference in the QoL between the 2 groups. At 24 weeks (T2), after adjusting for the baseline scores, participants who received the MGCS program plus routine care exhibited a significant improvement in QoL compared with participants who only received routine care (*P*=.04; *d*=0.30; adjusted mean difference 4.77, 95% CI 0.12-9.41; Table 3). No significant differences were found in symptom distress and social support between the 2 groups at 12 weeks (T1) and 24 weeks (T2) after adjusting for the baseline corresponding scores. There were significant time × group interaction effects for uncertainty in illness (*F*_1,165_=10.937; *P*=.001). Time effects were found for QoL (*F*_1,165_=10.192; *P*=.002). The time × group interactions and time effects were not significant for symptom distress or social support. Figure 2 presents a graphical representation of the mean changes in the 4 measurement outcomes.

#### Usage Data of the MGCS Program

The MGCS program usage varied considerably. During the 24-week intervention, the mean of the total usage duration was 85.23 minutes (SD 196.24; maximum 1144.60; median 16.53, IQR 2.12-75.20), and the mean of the total log-in frequency was 67.90 times (SD 106.01; maximum 565; median 21, IQR 4-94). The weekly topics and discussion center modules were most commonly used by participants. The health consultation module was the least used module in our study. Table 4 shows the usage duration and log-in frequency of the entire MGCS program and each of the 4 modules. The association between MGCS usage data and health outcomes was not found in our study.

**Table 4:** Usage duration and log-in frequency of the mobile app for gynecologic cancer support program for participants during the 24-week intervention (n=84).

Program and modules	Usage duration (minutes)			Log-in frequency (times)		
	Mean (SD)	Median (IQR)	Maximum	Mean (SD)	Median (IQR)	Maximum
						
Entire mobile app for gynecologic cancer support program	85.23 (196.24)	16.53 (2.12-75.20)	1144.60	67.90 (106.01)	21 (4-94)	565
Weekly topics	32.28 (97.72)	2.83 (0.38-22.18)	665.02	21.37 (36.08)	4 (1-19)	171
Emotional care	22.45 (129.10)	0.13 (0.00-1.83)	1053.47	9.15 (35.34)	1 (0-6)	277
Discussion center	28.51 (94.21)	2.88 (0.20-19.98)	744.38	30.55 (60.35)	4 (1-26)	282
Health consultation	1.98 (4.83)	0.10 (0.00-1.00)	24.78	6.82 (16.18)	1 (0-8)	107

## Discussion

### Principal Results

The strengths of this study include the clinical importance of an app for supporting patients with GC and the theory-derived intervention as well as the methodological rigor in collecting and analyzing data. The MGCS program is the first expert-led app-based program designed in China for patients with GC receiving chemotherapy. The MGCS program illustrates how an app-based support program can be incorporated into routine care to promote health outcomes for patients with GC receiving chemotherapy.

Our trial shows that the MGCS program significantly decreased the uncertainty in patients with GC at 24 weeks. This is consistent with that reported in another study, which found that a mobile navigation program significantly decreased the uncertainty in patients with colorectal cancer [22]. During chemotherapy, patients with GC may feel ambiguous about what to expect from their illness and treatment because they are not provided with clear, credible, and sufficient information [14]. Patients with GC may have difficulty in understanding their illness and treatments because of the complexity of treatment outcomes and side effects [14]. Even at the completion of chemotherapy, patients with GC feel significant uncertainty about their prognosis and may continue to seek prognostic information [42]. In the MGCS program, the 24 topics were validated by multidisciplinary health care professionals and updated each week according to the treatment course and chemotherapy regimen. The appraisal and coping strategies they learnt, the access to peer support, and the health professional consultation may have enhanced the knowledge of patients with GC on chemotherapy-induced side effects and helped them achieve greater clarity about health and treatment outcomes. Our trial shows that Mishel uncertainty in illness theory has the potential to guide the design of an app-based program to decrease uncertainty in illness.

Our results show that the MGCS program significantly improved the QoL of patients with GC at 24 weeks. Minimally important clinical differences with values over 3-7 in the FACT-G scale have been established as clinically meaningful improvements in QoL [43]. In our study, the between-group mean differences value in FACT-G scale was 4.77, which indicated clinically meaningful findings. Sato [44], in a single-arm study, found that a telenursing program for 30 men with prostate cancer positively promoted their QoL. That program included 2 modules: information entry by men about their physical symptoms and complications after surgery and responses from medical staff to offer personalized nursing support and emotional support via email and web-based chat platforms [44]. The health consultation module in the MGCS program has similar functionality as the telenursing program. Patients with GC could ask any question about health concerns, and the health care team provided credible information and emotional support. The sense of security through communication with an experienced health care team was thought to contribute greatly to the improvement of emotional well-being [44]. Kinner and colleagues [45] conducted a single-arm pilot study of an internet-based 10-week stress management program involving daily relaxation and reflection, weekly content overviews, and video conferences. This program recruited 19 patients with ovarian cancer and reported an improved QoL [45]. The emotional care module in the MGCS program also provided relaxation and distraction strategies, which might contribute to an improvement in perceived stress, ultimately affecting the QoL. Compared to previous studies [44,45], our trial had a more robust design and included a greater variety of physical, psychological, and emotional support. Our study demonstrates that app-based support programs create an easily accessible and convenient platform for information provision, communication, and support, thereby improving the QoL of patients with GC.

Our trial shows that the MGCS program did not significantly improve the symptom distress and social support of patients with GC at 24 weeks. In the MGCS program, symptom management strategies were provided, but the practices of the strategies were not tracked or monitored. Patients with GC may not practice these strategies or may get tired of doing the same exercises strategies over time [46], resulting in a lack of improvement in their symptom distress over the course of the program. This should be addressed in future studies. The mean score of social support at baseline in both groups indicated existing good social support [47]. A lack of increase in this score was, therefore, not considered problematic.

We found no significant differences in any of the health outcomes between the intervention and control groups at 12 weeks. During early chemotherapy cycles, patients with GC experience high levels of physical and psychological distress, which may limit their ability or desire to read, interact with others, and practice coping strategies [48]. Future studies could explore some strategies such as real-time monitoring and management system or synchronous videoconferences to strengthen the intervention usage and efficacy to better assist those in need [49,50]. Engagement with all modules of the MGCS program was not consistent. The average log-in frequency was 68 times, which means that patients with GC logged into MGCS 2-3 times per week during the 24-week intervention. Patients with GC spent approximately 4 minutes reading or chatting in MGCS each week. The big difference between mean and median in the log-in frequency and usage duration indicated usage polarization among patients with GC. This is consistent with results from our previous study on a mobile app program for Chinese patients with breast cancer undergoing chemotherapy [35]. The usage polarization of the MGCS program can be partially explained by the variation of reading levels and age of patients with GC, with 62% of patients with GC reading at junior high level or below and 18% of patients with GC aged 60 years or older in our program. There are differences in understanding and familiarity with digital devices based on patients’ education level and age. Patients’ low education level and old age were found to be positively associated with their engagement in mobile health programs [51,52]. Our findings show that patients with GC did not use all the MGCS modules; they preferred the weekly topics and discussion center modules as their information resource and emotional support. Patients with GC did not use the health consultation module very often, which may indicate that they did not feel comfortable with web-based consultations. We did not find an association between MGCS usage data and health outcomes. Future studies are warranted to explore strategies to encourage engagement of patients with GC with the app and further assess the impact of enhanced program engagement on health outcomes.

### Limitations

This trial has several limitations. We only recruited patients with 3 types of GCs, while those with other GCs (eg, vulvar cancer) were excluded. Patients with GC recruited from the 2 participating university-affiliated hospitals may not represent those in the hospitals located in rural or remote areas. Implementing the intervention may exclude patients who were unfamiliar with technology and not comfortable with technology, which might further hinder generalizability. The evaluation data are more likely to reflect only those patients with a strong interest in psychosocial interventions, which may lead to positive bias. Additionally, the information on patients' treatment regimen changes during the trial were not collected, and its impacts on the outcomes could not be known. We only followed up patients with GC until the completion of our intervention at 24 weeks. Some researchers have shown that web-based cognitive behavioral therapy interventions have a positive effect on the improvement of QoL and decrease of symptoms at 1-year and 3-year follow-ups [53]. Thus, we were not able to know whether the effects of the MGCS program sustain after our intervention or the duration of the effects. Future studies are warranted to recruit patients from a variety of demographic and medical conditions, record the changes in the treatment regimen, and extend the follow-up time to test the long-term effects. Moreover, the motivation to access apps and posts in the module of discussion center may change depending on what stage of chemotherapy a patient is in, especially during or after the chemotherapy. MGCS has not been designed to track the usage data of patients with GC on a dynamic basis, and the posts reflecting patients’ concerns have not been collected, which means that it is not possible to know the change pattern of MCCS engagement and patients’ concerns. Future studies are recommended to collect the dynamic usage statistics and posts to specify the minimal requirements of participant usage statistics, set the criteria for adherence, and gain insight of patients’ concerns during the different courses of treatment for better design and implementation of app-based interventions.

### Conclusions

This trial demonstrates the efficacy of an app-based program to decrease uncertainty in illness and improve the QoL of patients with GC receiving chemotherapy. This trial illustrates how an app-based support program can be incorporated into clinical practice. Translating the intervention into clinical practice can be strengthened by establishing optimal dosage or by adapting for the larger cancer population online. This trial suggests that allocation of greater resources to mobile health interventions in clinics is warranted.

## Appendix

Multimedia Appendix 1 CONSORT-EHEALTH (V1.6.1) checklist.

## Abbreviations

CONSORT-EHEALTH: Consolidated Standards of Reporting Trials of Electronic and Mobile Health Applications and Online Telehealth
FACT-G: Functional Assessment of Cancer Therapy-General
GC: gynecologic cancer
MDASI: MD Anderson Symptom Inventory
MGCS: mobile app for gynecologic cancer support
MSPSS: Multidimensional Scale of Perceived Social Support
MUIS-A: Mishel Uncertainty in Illness Scale for Adults
QoL: quality of life

## Data Availability

The data sets generated and analyzed during this study are available in the Mendeley repository: 10.17632/cmn7zfbwby.2

## References

[1] Ferlay J, Colombet M, Soerjomataram I, Parkin DM, Piñeros Marion, Znaor A, Bray F (2021). Cancer statistics for the year 2020: An overview. Int J Cancer. Online ahead of print.

[2] Estimated age-standardized incidence and mortality rates (World) in 2020, China, females, all ages. Cancer Today.

[3] Reed NS, Sadozye AH (2016). Update on chemotherapy in gynaecological cancers. The Obstetric & Gynaecologis.

[4] Walton LM, Reeve J, Brown PM, Farquhar CM (2010). Gynaecologic cancer patients' needs and experiences of supportive health services in New Zealand. Psychooncology.

[5] Lopez AJ, Butow PN, Philp S, Hobbs K, Phillips E, Robertson R, Juraskova I (2019). Age-related supportive care needs of women with gynaecological cancer: A qualitative exploration. Eur J Cancer Care (Engl).

[6] Hodgkinson K, Butow P, Hunt GE, Wyse R, Hobbs KM, Wain G (2007). Life after cancer: couples' and partners' psychological adjustment and supportive care needs. Support Care Cancer.

[7] McDowell L, Rischin D, Gough K, Henson C (2022). Health-related quality of life, psychosocial distress and unmet needs in older patients with head and neck cancer. Front Oncol.

[8] Zeng Y, Cheng AS, Liu X, Chan CC (2017). Title: Cervical cancer survivors' perceived cognitive complaints and supportive care needs in mainland China: a qualitative study. BMJ Open.

[9] Saeidzadeh S, Kamalumpundi V, Chi N, Nair R, Gilbertson-White S (2021). Web and mobile-based symptom management interventions for physical symptoms of people with advanced cancer: A systematic review and meta-analysis. Palliat Med.

[10] Number of mobile internet users in China from 2019 to 2023 with a forecast until 2028 (in millions). Statista.

[11] Lin H, Ye M, Chan SW, Zhu J, He H (2020). The effectiveness of online interventions for patients with gynecological cancer: An integrative review. Gynecol Oncol.

[12] Aldaz BE, Hegarty RSM, Conner TS, Perez D, Treharne GJ (2019). Is avoidance of illness uncertainty associated with distress during oncology treatment? A daily diary study. Psychol Health.

[13] Mishel MH, Braden CJ (1988). Finding meaning: antecedents of uncertainty in illness. Nurs Res.

[14] Kalke K, Studd H, Scherr CL (2021). The communication of uncertainty in health: A scoping review. Patient Educ Couns.

[15] Haisfield-Wolfe ME, McGuire DB, Soeken K, Geiger-Brown J, De Forge B, Suntharalingam M (2012). Prevalence and correlates of symptoms and uncertainty in illness among head and neck cancer patients receiving definitive radiation with or without chemotherapy. Support Care Cancer.

[16] Lee I, Park C (2020). The mediating effect of social support on uncertainty in illness and quality of life of female cancer survivors: a cross-sectional study. Health Qual Life Outcomes.

[17] Verduzco-Aguirre HC, Babu D, Mohile SG, Bautista J, Xu H, Culakova E, Canin B, Zhang Y, Wells M, Epstein RM, Duberstein P, McHugh C, Dale W, Conlin A, Bearden J, Berenberg J, Tejani M, Loh KP (2021). Associations of uncertainty with psychological health and quality of life in older adults with advanced cancer. J Pain Symptom Manage.

[18] Zhang Y (2017). Uncertainty in illness: theory review, application, and extension. Oncol Nurs Forum.

[19] Lin H, Chan SW, Ye M, Wang Y, Liu H, Li M, Liu S, Zhu J (2021). A multi-centre randomized controlled trial of mobile gynaecological cancer support program for patients with gynaecological cancer undergoing chemotherapy: Study protocol. J Adv Nurs.

[20] Eysenbach G, CONSORT-EHEALTH Group (2011). CONSORT-EHEALTH: improving and standardizing evaluation reports of web-based and mobile health interventions. J Med Internet Res.

[21] Brashers DE, Basinger ED, Rintamaki LS, Caughlin JP, Para M (2017). Taking control: the efficacy and durability of a peer-led uncertainty management intervention for people recently diagnosed with HIV. Health Commun.

[22] Kim K, Park W (2019). Effects of mobile navigation program in colorectal cancer patients based on uncertainty theory. J Korean Acad Nurs.

[23] Research Randomizer (Version 4.0).

[24] Efird J (2011). Blocked randomization with randomly selected block sizes. Int J Environ Res Public Health.

[25] Chemotherapy side effects. American Cancer Society.

[26] Sheu S, Hwang SL (1996). Validation of Chinese version of Mishel's uncertainty in illness scale. Clin Nurs Res (Taiwan).

[27] Mishel MH, Sorenson DS (1991). Uncertainty in gynecological cancer: a test of the mediating functions of mastery and coping. Nurs Res.

[28] Lee Y, Wang H, Li C, Shiao A, Tu T (2014). Exploring the effectiveness of a health education program on the stimuli frame and on uncertainty in patients with sudden hearing loss. Hu Li Za Zhi.

[29] Cheung YB, Goh C, Wee J, Khoo KS, Thumboo J (2009). Measurement properties of the Chinese language version of the functional assessment of cancer therapy-general in a Singaporean population. Ann Acad Med Singap.

[30] Barnes D, Rivera R, Gibson S, Craig C, Cragun J, Monk B, Chase D (2018). The utility of patient reported data in a gynecologic oncology clinic. Gynecol Oncol Res Pract.

[31] Edianto D, Yaznil MR, Chartyansari AA, Effendi IH (2019). Assessment of the quality of life for gynecologic cancer patients using Functional Assessment of Cancer Therapy-General (Fact-G) questionnaire at Haji Adam Malik hospital. Open Access Maced J Med Sci.

[32] Tsai W, Lu Q (2018). Perceived social support mediates the longitudinal relations between ambivalence over emotional expression and quality of life among Chinese American breast cancer survivors. Int J Behav Med.

[33] Wang XS, Wang Y, Guo H, Mendoza TR, Hao X, Cleeland CS (2004). Chinese version of the MD Anderson Symptom Inventory: validation and application of symptom measurement in cancer patients. Cancer.

[34] Armbruster SD, Sun CC, Westin SN, Bodurka DC, Ramondetta L, Meyer LA, Soliman PT (2018). Prospective assessment of patient-reported outcomes in gynecologic cancer patients before and after pelvic exenteration. Gynecol Oncol.

[35] Zhu J, Ebert L, Liu X, Wei D, Chan SW (2018). Mobile breast cancer e-support program for Chinese women with breast cancer undergoing chemotherapy (part 2): multicenter randomized controlled trial. JMIR Mhealth Uhealth.

[36] Zhou K, Li H, Wei X, Yin J, Liang P, Zhang H, Kou L, Hao M, You L, Li X, Zhuang G (2015). Reliability and validity of the multidimensional scale of perceived social support in Chinese mainland patients with methadone maintenance treatment. Compr Psychiatry.

[37] Calderón Caterina, Ferrando PJ, Lorenzo-Seva U, Gómez-Sánchez David, Fernández-Montes Ana, Palacín-Lois Maria, Antoñanzas-Basa Mónica, Rogado J, Manzano-Fernández Aránzazu, Ferreira E, Asensio-Martínez Elena, Jiménez-Fonseca Paula (2021). Multidimensional Scale of Perceived Social Support (MSPSS) in cancer patients: psychometric properties and measurement invariance. Psicothema.

[38] (2017). Downloading IBM SPSS statistics 25. IBM Corp.

[39] Owen SV, Froman RD (1998). Uses and abuses of the analysis of covariance. Res Nurs Health.

[40] Moher D, Hopewell S, Schulz KF, Montori V, Gøtzsche Peter C, Devereaux PJ, Elbourne D, Egger M, Altman DG, CONSORT (2012). CONSORT 2010 explanation and elaboration: updated guidelines for reporting parallel group randomised trials. Int J Surg.

[41] Olejnik S, Algina J (2000). Measures of effect size for comparative studies: applications, interpretations, and limitations. Contemp Educ Psychol.

[42] Han PKJ, Gutheil C, Hutchinson RN, LaChance JA (2020). Cause or effect? The role of prognostic uncertainty in the fear of cancer recurrence. Front Psychol.

[43] Bauersfeld SP, Kessler CS, Wischnewsky M, Jaensch A, Steckhan N, Stange R, Kunz B, Brückner Barbara, Sehouli J, Michalsen A (2018). The effects of short-term fasting on quality of life and tolerance to chemotherapy in patients with breast and ovarian cancer: a randomized cross-over pilot study. BMC Cancer.

[44] Sato D (2020). Effectiveness of telenursing for postoperative complications in patients with prostate cancer. Asia Pac J Oncol Nurs.

[45] Kinner EM, Armer JS, McGregor BA, Duffecy J, Leighton S, Corden ME, Gauthier Mullady J, Penedo FJ, Lutgendorf SK (2018). Internet-based group intervention for ovarian cancer survivors: feasibility and preliminary results. JMIR Cancer.

[46] Kwekkeboom K, Zhang Y, Campbell T, Coe CL, Costanzo E, Serlin RC, Ward S (2018). Randomized controlled trial of a brief cognitive-behavioral strategies intervention for the pain, fatigue, and sleep disturbance symptom cluster in advanced cancer. Psychooncology.

[47] Zimet GD, Dahlem NW, Zimet SG, Farley GK (1988). The multidimensional scale of perceived social support. Journal of Personality Assessment.

[48] Hsu H, Tsai S, Wu S, Jeang S, Ho M, Liou W, Chiang A, Chang T (2017). Longitudinal perceptions of the side effects of chemotherapy in patients with gynecological cancer. Support Care Cancer.

[49] Graetz I, Anderson JN, McKillop CN, Stepanski EJ, Paladino AJ, Tillmanns TD (2018). Use of a web-based app to improve postoperative outcomes for patients receiving gynecological oncology care: A randomized controlled feasibility trial. Gynecol Oncol.

[50] Classen CC, Chivers ML, Urowitz S, Barbera L, Wiljer D, O'Rinn S, Ferguson SE (2013). Psychosexual distress in women with gynecologic cancer: a feasibility study of an online support group. Psychooncology.

[51] Crouch E, Gordon NP (2019). Prevalence and factors influencing use of internet and electronic health resources by middle-aged and older adults in a US health plan population: cross-sectional survey study. JMIR Aging.

[52] Zhu H, Chen X, Yang J, Wu Q, Zhu J, Chan SW (2020). Mobile breast cancer e-support program for Chinese women with breast cancer undergoing chemotherapy (part 3): secondary data analysis. JMIR Mhealth Uhealth.

[53] Andersson G, Hesser H, Veilord A, Svedling L, Andersson F, Sleman O, Mauritzson L, Sarkohi A, Claesson E, Zetterqvist V, Lamminen M, Eriksson T, Carlbring P (2013). Randomised controlled non-inferiority trial with 3-year follow-up of internet-delivered versus face-to-face group cognitive behavioural therapy for depression. J Affect Disord.

